# Exposure of South African Abattoir Workers to *Coxiella burnetii*

**DOI:** 10.3390/tropicalmed7020028

**Published:** 2022-02-16

**Authors:** Liesl De Boni, Sumaya Mall, Veerle Msimang, Alex de Voux, Jennifer Rossouw, John Frean

**Affiliations:** 1 South African Field Epidemiology Training Programme, Johannesburg 2192, South Africa; alex.devoux@gmail.com; 2 Division of Epidemiology and Biostatistics, School of Public Health, University of the Witwatersrand, Johannesburg 2193, South Africa; sumaya.mall@wits.ac.za; 3 National Institute for Communicable Diseases, Division of the National Health Laboratory Service, Johannesburg 2192, South Africa; veerlem@nicd.ac.za (V.M.); jennyr@nicd.ac.za (J.R.); johnf@nicd.ac.za (J.F.); 4 Wits Research Institute for Malaria, University of the Witwatersrand, Johannesburg 2193, South Africa

**Keywords:** Q fever, *Coxiella burnetii*, Q fever prevalence, seroprevalence, abattoir workers, meat workers, slaughterhouse workers

## Abstract

Abattoir workers may contract Q fever by inhalation of *Coxiella burnetii* bacteria in aerosols generated by slaughtering livestock, or in contaminated dust. We estimated the seroprevalence of *C. burnetii* and examined the associated factors in a survey of South African abattoir workers. *Coxiella burnetii* seropositivity was determined by detection of IgG antibodies against *C. burnetii* phase II antigen. Logistic regression, adjusted for clustering and sampling fraction, was employed to analyze risk factors associated with *C. burnetii* seropositivity. Among 382 workers from 16 facilities, the overall seroprevalence was 33% (95% confidence interval (CI): 28–38%) and ranged from 8% to 62% at the facility level. Prolonged contact with carcasses or meat products (odds ratio (OR): 4.6, 95% CI: 1.51–14.41) and prior abattoir or butchery work experience (OR: 1.9, 95% CI: 1.13–3.17) were associated with *C. burnetii* seropositivity. In contrast, increasing age and livestock ownership were inversely associated. Precautions to protect abattoir personnel from Q fever are discussed.

## 1. Introduction

Q fever is a zoonotic bacterial disease caused by *Coxiella burnetii* and is prevalent in most countries [1,2]. Predominantly an occupational disease, it affects people working with animals (farmers, abattoir workers, and veterinary staff) or the bacteria (laboratory personnel) [2]. Normally, sporadic cases are observed in endemic rural scenarios, but outbreaks may occur when immune-naïve people encounter *C. burnetii* [2]. A large urban outbreak occurred in the Netherlands in the period 2007–2010, with approximately 4000 human cases acquired from infected dairy goat herds in a densely populated region [3]. *Coxiella burnetii* has an infectious spore-like form that is environmentally stable and can persist in soil for years to spread by the airborne route [4]. Transmission to humans occurs by inhalation of aerosols or dust contaminated with the bacteria. Infected animals shed *C. burnetii* in their excrements and the products of abortion, which is often the only sign of infection in animals [2,4,5]. Acute Q fever in people is asymptomatic in 60% of cases and presents as a mild influenza-like syndrome in the remaining individuals, but may progress to more serious complications including pneumonia, hepatitis, meningitis, osteomyelitis, and obstetric problems [1,5]. Furthermore, patients who have cleared an acute *C. burnetii* infection may develop chronic fatigue syndrome that can persist for months. Occasionally (in <5% of cases), the bacteria can survive in monocytes to cause illness later in life, known as latent infection, and this can manifest as infective endocarditis or vascular prosthesis infection [1,2,5].

After exposure to *C. burnetii*, immunoglobulins M and G (IgM and IgG) against phase II antigen are first to rise, almost simultaneously [6]. Analysis of the Netherlands outbreak data suggested that phase II IgG appeared first, reached the highest levels and persisted for the longest time [7]. Hence, phase II IgG is the preferred antibody marker for previous *C. burnetii* infection or exposure. Asymptomatic seroconversion is common, occurring among approximately 60% of cases [1,5]. Infection in childhood is often mild or asymptomatic [2] and continuous occupational re-exposure causes high IgG antibody levels to persist [8]. Thus, a group of individuals seropositive for phase II IgG would be a mixture of those that had clinical illness of variable severity at some point and those that never had symptoms.

There are limited human seroprevalence studies of *C. burnetii* on the African continent, which yielded varying results [2,9] and the prevalence of Q fever is likely underestimated for several reasons. A high proportion of cases are asymptomatic or mild with non-specific symptoms, and *C. burnetii* tests are not routinely requested or available [2,10]. The disease is generally not a notifiable condition, going unreported in both humans and animals [6]. Recorded human seroprevalence in Africa ranged between 1% in Chad and 37% in Zimbabwe [11,12], with the infection being present in Mali, Burkina Faso, Nigeria, Central African Republic, Egypt, Namibia, Algeria and rural Senegal [2,11,12]. Relatively high seroprevalence, of 8–17%, was reported among children in Ghana, Niger and the Gambia [11]. Reported seroprevalence in African domestic animals was high, with 4–55% in cattle, 13–24% in goats, 11–33% in sheep, 23% in dogs and 70–80% in camels [9,11].

The first human Q fever cases in South Africa were documented in 1950 [13,14]. Subsequent surveys found it to be common [10], and it was assumed that most rural-living South Africans had acquired the infection, and immunity, during childhood. Illness was mainly observed among newly arrived immigrants and urban residents [15]. By 1987, *C. burnetii* had been detected in fetal and placental tissues of cattle and sheep on farms across the country [16], and seroprevalence of 8% was reported in 8900 cattle in the former Transvaal Province [17]. Two more recent surveys found 61% and 33% seroprevalence among healthy people working in close contact with animals in Mpumalanga Province in 2012 and 2014, respectively [18] (Msimang V, 2014. National Institute for Communicable Diseases, personal communication).

Q fever was first detected in abattoir workers in Australia in 1935 [19] and they were recognized as a high-risk group for Q fever ever since. Progress was made to protect abattoir workers in Australia from Q fever, in the form of a targeted vaccination program introduced in 2002 [20]. Q fever notification rates declined by 50% after the program started [21]. In the Netherlands, the same vaccine was registered for specific risk groups, i.e., patients with heart valve disorders, from 2011 [22]. We know from Australian data that the duration of Q fever illness can be protracted, with a median of 3 weeks for sickness and 12 weeks to full recovery [23]. This prolonged duration of illness and recovery, and that exposure commonly occurs in abattoir environments, demonstrates the importance of *C. burnetii* as an occupational hazard. In South Africa, there is no compensation for Q fever acquired at work as it is not a prescribed occupational disease (Employees’ Compensation Act, Act No. 30 of 1941).

A wide range of *C. burnetii* seroprevalence, 9–30%, was recorded among abattoir workers in South Korea, Japan, Iran, Scotland, and Brazil [24,25,26,27,28,29,30]. A meta-analysis of 19 papers estimated an overall seroprevalence of 26% (95% confidence interval (CI): 18–35%) among abattoir workers in Australia, Brazil, Canada, Ethiopia, Iran, Japan, Spain, Trinidad, Turkey, the United Kingdom, and the United States. Slaughtering livestock (cattle, sheep and goats) was reported as the main risk factor for seropositivity and development of symptomatic disease [31]. Similar levels of pre-existing *C. burnetii* immunity due to prior infection were found in abattoir workers in New South Wales (29%, *n* = 485) and Queensland (34%, *n* = 1751) by prescreening tests for the Australian national vaccination program [20]. Thus, reasonably high seroprevalence has been reported for *C. burnetii* in abattoir workers globally.

Q fever disease has been recognized in South Africa since at least 1950 [13,14] and it is a well-described problem among abattoir workers in other countries. Nevertheless, little new information has been produced in South Africa, particularly in abattoir workers. We set out to estimate the seroprevalence of *C. burnetii* and explore factors associated with prior infection among abattoir workers in the Free State and Northern Cape provinces of South Africa.

## 2. Materials and Methods

A serological survey was conducted to investigate abattoir workers for previous infection with *C. burnetii*, Rift Valley fever, Crimean–Congo hemorrhagic fever and brucellosis. This is a cross-sectional study of the findings pertaining to *C. burnetii*.

The data collection procedure entailed several steps. First, all functioning abattoirs located within the study area were sampled from March to May of 2018. At each participating facility, workers were selected using convenience sampling. All consenting workers could participate, but the number sampled depended on the time available. At small abattoirs, most of the workers participated. At large busy facilities, it was not possible to sample all willing workers, so those who performed slaughter and/or handled carcasses were prioritized and fewer other workers were sampled. This purposive selection was not performed systematically. The participants completed individual electronic questionnaires on a mobile device. This questionnaire collected individual demographic, health-related, knowledge, practices, and exposure data. Participants were mainly abattoir workers but included the management when they were willing to participate. A professional nurse collected a venous blood specimen in a serum-separator tube from each participant. Each owner/manager provided information about the abattoir facility via a separate electronic questionnaire. The sera derived from the blood specimens were refrigerated and transported to the Centre for Emerging Zoonotic and Parasitic Diseases at the National Institute for Communicable Diseases (NICD), a division of the National Health Laboratory Service (NHLS), for storage at −20.0 °C until testing. A commercial enzyme-linked immunosorbent assay test was used (Vircell, Granada, Spain) to detect IgG antibody against the *C. burnetii* phase II Nine Mile (ATCC VR-616) antigen as per the manufacturer’s instructions. All equivocal specimens were retested. The serological results and questionnaire response data analyzed here have been provided in the Supplementary Material (Table S1).

The participant number was used to merge the test results with the cleaned questionnaire data received from the abattoir survey study group using Stata version 15 (StataCorp, College Station, TX, USA). Equivocal serological results were excluded from the analysis. Exposure to tick bites and performance of specific work activities were transformed into binary variables. Education levels were grouped to reflect the South African norm. Total staff employed per facility, average animals slaughtered daily, and number of years established were used to create categorical variables for abattoir size, throughput, and lifespan. A map of abattoir locations was made for this paper using Esri ArcGIS 10.2, with the 2016 provincial boundary shapefile from the SA Municipal Demarcation Board (https://dataportal-mdb-sa.opendata.arcgis.com/, accessed on 20 July 2021).

To account for the clustering and sampling fraction of participants within abattoir facilities, we used the linearized variance estimator function, based on a first-order Taylor series linear approximation, to estimate the apparent seroprevalence with confidence intervals [32]. The ‘svy-set’ command was used to specify the abattoir identifier as the cluster variable and ‘pweight’ to specify the sampling fraction. For comparison, we also produced a true seroprevalence estimate, which is the apparent seroprevalence adjusted by the test sensitivity and specificity [33]. The sensitivity and specificity of the test used were 95% and 97%, respectively. We used logistic regression analysis to examine associations between numerous exposures of interest and seropositivity. This logistic regression analysis used fixed effects. We adjusted for clustering and sampling fraction by abattoir facility by using the ‘svy’ prefix command after the abattoir identifier and sampling fraction had been specified with ‘svy-set’. All variables with *p*-values < 0.2 in the univariable analysis were considered in the multivariable analysis. Backward elimination, with a *p*-value threshold of 0.1, was used to remove unsuitable exposure variables in three stages. Starting with sociodemographic variables, then facility-level factors, then individual factors, exposure variables qualifying at each stage were carried into the subsequent model. To control for the effect of previous work experience in the abattoir/butchery industry, that factor was kept in the final model regardless of the statistical value. Exposure variables with a *p*-value < 0.05 in the final model were considered statistically significant. The goodness-of-fit test which estimates the F-adjusted mean residual test was used to assess the model fit because it is more appropriate for logistic regression models fitted using survey data [34].

## 3. Results

### 3.1. Participating Abattoir Workers

**Figure 1:**
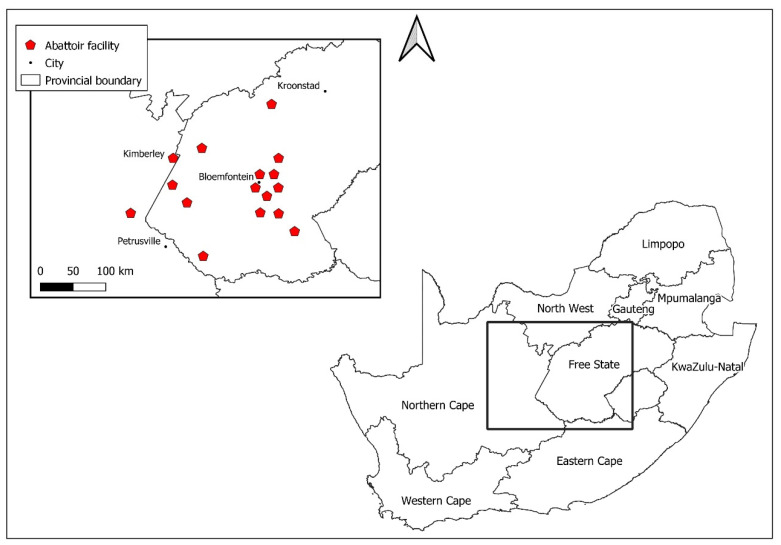
Locations of abattoir facilities sampled in the abattoir survey, South Africa, 2018.

Overall, 382 abattoir workers from 16 facilities were sampled (Figure 1), equating to approximately one third of the total number of abattoir workers recorded in the area during this study, ~1350. The individual refusal rates were low, and only one facility declined to participate. The facility sizes varied from 6 to 520 employees, with relatively lower participation rates among larger facilities. The median age of participants was 35 years (interquartile range 29–42 years). Most participants were male and had attained secondary school education (Table 1).

The majority of participants were employed full time (96%, 351/364) and worked inside the abattoir (93%, 343/369). Those not working inside the abattoir worked either in administration, management, or as drivers. The median period of employment at the facility was 5 years (interquartile range: 3–10 years). Almost one-third of participants had prior abattoir/butchery work experience (27%, 101/368) and 17% (62/368) had prior work experience in farming. Some individuals reported owning livestock personally (16%, 58/368), with most of them owning sheep/goats (7%, 24/368), cattle only (6%, 21/368), or a mixture of livestock (4%, 13/368). The most frequent work tasks performed were cleaning abattoir equipment (77%, 293/382), cleaning slaughter areas (74%, 282/382), slaughter/evisceration/carcass dressing (55%, 209/382) and cleaning other areas (55%, 209/382). Most of the workers sampled performed multiple tasks and fewer than five reported performing a single task exclusively. Almost all the participants worked in high-throughput facilities (93%, 355/382) and more worked in large facilities (67%, 256/382) than small facilities. Sheep were processed at all the facilities, but participants also processed cattle (82%, 315/382), pigs (24%, 90/382) and other species such as goats, poultry and wild antelope (<3%). One-quarter of participants reported receiving medical treatment for a chronic condition (25%, 91/366). Most abattoir facilities required staff to wear gloves (85%, 324/382), masks (71%, 271/382) or goggles (67%, 257/382), but no data were collected on compliance.

### 3.2. Coxiella burnetii Seroprevalence Estimates

Out of 359 valid test results, 109 were positive, producing an apparent seroprevalence of 33% (95% CI: 28–38%), which was adjusted for clustering and sampling fraction. The apparent seroprevalence differed widely between facilities, at a range of 8–62%. The true seroprevalence, which considered the test characteristics, was 30% (95% CI: 25–35%) (Table 2).

### 3.3. Correlates of C. burnetii Seropositivity

**Figure 2:**
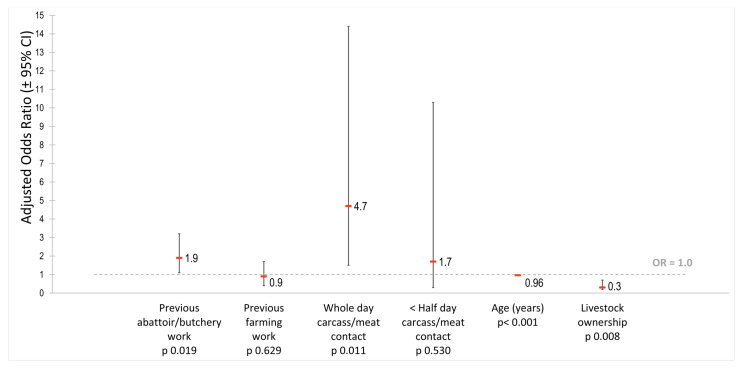
Factors significantly associated with *Coxiella burnetii* seropositivity in the multivariable logistic regression analysis of the abattoir survey, South Africa, 2018.

The results of the univariable and multivariable analyses are shown (Table 3 and Figure 2). Participants with prior abattoir/butchery work experience (*n* = 94) were 1.9-fold (95% CI: 1.1–3.2) as likely to be seropositive than those without previous work experience in the meat or farming industry. Compared to those who reported spending less than an hour in contact with carcasses or meat products on a typical workday (*n* = 17), abattoir workers who reported spending the whole day in contact (*n* = 299) were 4.7-fold (95% CI: 1.5–14.4) as likely to be seropositive than those spending less than an hour per day. In contrast, age appeared to have a very small protective effect. For every year increase in age, the odds of being seropositive were 0.96-fold (95% CI: 0.94–0.98). Personal ownership of livestock also demonstrated a protective effect with livestock owners having 0.3-fold (95% CI: 0.2–0.7) the likelihood of being seropositive compared to non-owners. The post-regression goodness-of-fit test found the final model to be adequate.

## 4. Discussion

This serological survey of *C. burnetii* among South African abattoir workers yielded several important findings. Seroprevalence indicated previous exposure to *C. burnetii* in one-third of sampled workers. Several factors associated with *C. burnetii* seropositivity, namely prior experience working at an abattoir/butchery and prolonged contact with carcasses or meat products, were identified. In contrast, increasing age and livestock ownership were inversely associated.

Though the seroprevalence estimates were similar, the true seroprevalence estimate (30%) was more conservative than the apparent estimate (33%). The seroprevalence in abattoir workers reported here is equal to or higher than that documented in similar serological studies of abattoir workers, which ranged from 9% to 30% [24,25,26,27,28,29,30]. Between-country comparisons are difficult due to varying population characteristics, sample sizes, study designs and serological tests used [9,11]. However, a meta-analysis reported a lower overall seroprevalence of 26% [31]. Our relatively high seroprevalence estimate is comparable with what was observed in abattoir workers tested in New South Wales and Queensland before the Australian Q fever vaccination program was implemented in 2002 [20]. The seroprevalence may have been over-estimated in this study due to the participant selection method in which workers performing slaughter and/or handling carcasses were preferentially sampled at some facilities.

Working with carcasses or meat products emerged as a risk factor for *C. burnetii* seropositivity in this study in two ways. First, abattoir workers with previous experience working in the meat industry (*n* = 94) were 1.9-fold as likely to be seropositive than those who did not have work experience in either the meat or farming sector. Second, participants who reported spending the whole day in contact with carcasses or meat products were 4.7-fold as likely to have *C. burnetii* antibodies than those who spent less than an hour in contact on a typical workday. Abattoir workers are a high-risk group for Q fever since it was first recognized in Australian abattoir workers in 1935 [19], the World Health Organization classified it as an occupational zoonosis in this group in 1979 [21], and numerous outbreaks have been reported in this setting [24,35,36,37]. Performing slaughter [38] or evisceration [28], working with animal skins [19] and contact with cattle blood near the mouth [27] have been described as risk factors for *C. burnetii* exposure. One survey of Brazilian slaughterhouse employees found higher seroprevalence for workers in the livestock holding area (40%, 2/5) and those performing slaughter (36%, 26/73), compared to handling meat for deboning (20%, 7/35) or working in the sausage section (14%, 2/14) [25]. These findings from observational studies and outbreaks describe contact with live ruminants, their skins, viscera, or blood as likely mechanisms for *C. burnetii* transmission in the abattoir setting. In this study, most workers performed multiple duties with many involved in cleaning tasks, and none were confined to a certain section in the abattoir. This could explain why no specific work activity arose as a risk factor in the analysis. It is impossible to say whether the meat could be the actual source of exposure for these individuals using these data, but it is less likely, and no literature was found to support the notion. It seems more likely that participants were exposed to the bacterium when it was aerosolized as they worked, e.g., when herding livestock at the holding pens or cleaning already contaminated areas.

The two characteristics inversely associated with *C. burnetii* seropositivity were unexpected and difficult to explain. First, the analysis indicated lower seropositivity among livestock owners in this group. Two Iranian surveys reported higher *C. burnetii* seropositivity in at-risk people who also kept sheep/goats [39,40]. Further, the airborne presence of *C. burnetii* with a seasonal pattern coinciding with the goat kidding season in the Netherlands was shown, demonstrating the risk of infection to anyone living nearby goat farms at kidding time [41]. The effect of livestock ownership in this study may have occurred by chance, or there could be some other explanation. Livestock ownership may not have been an indicator for close contact or even proximity with livestock. More information is needed about the degree, duration, and nature of contact between these livestock owners and their animals to better understand the relationship. Second, increasing age was negatively associated with seropositivity, which also disagreed with the literature [42,43] but the effect was small. Normally, *C. burnetii* seroprevalence increases with age due to increased length of exposure and the persistence of IgG antibodies for years [7,8]. Further, continuous exposure to *C. burnetii* is expected in abattoir workers and this should result in persistently high antibody levels [8]. Perhaps work responsibilities change with increasing age, and this could result in less exposure to animals/carcasses at the abattoirs sampled, but more information is needed. The variability of age in these data was also low, with participants being in similar age groups, so this finding could be a random association.

Our findings suggest potential measures to protect abattoir workers from Q fever at work. Exposure probably occurred primarily by inhalation or close contact with aerosolized *C. burnetii.* We observed that most participants were required to wear personal protective equipment at work but could not assess the levels of compliance. Other studies showed that good hand hygiene [39], wearing gloves [44,45] and respiratory protection [2,46] prevented *C. burnetii* infections at high-risk workplaces. Therefore, personnel working closely with livestock, meat, or those involved in cleaning these areas should practice high-level hand hygiene and wear gloves, masks and/or protective eyewear, as appropriate for their duties. This has the added advantage of protecting them from other infectious zoonotic diseases. Abattoir staff should be educated about Q fever, trained in PPE use at work and to seek medical attention if they develop any febrile illness. Abattoir management can further reduce disease risk to personnel by only accepting healthy livestock from reputable sources. However, since outwardly healthy animals may still pose a *C. burnetii* risk, PPE will always be necessary. In the long term, controlling *C. burnetii* in animals is the best way to protect people, but it is unlikely to be prioritized as it is not a notifiable animal disease. More work is needed to describe the disease in South African livestock and people to understand the true disease burden and inform occupational safety measures.

The main limitations of this study are due to the cross-sectional study design, so causality cannot be inferred. However, the phase II IgG antibodies persist for at least 5 years [7] and the median employment time was 5 years. Abattoir workers may have been exposed elsewhere in the preceding 5 years, but as full-time employees it is likely that this was at work, and we adjusted for prior abattoir/butchery work experience. We could not analyze whether seropositivity differed by work area within the abattoir or whether performing informal slaughter outside of working hours played a role, as these data were not collected. Other limitations to consider arose from the diagnostic method. Serology assesses the humoral immune response only not cell-mediated immunity, and false positives due to cross-reactivity of antibodies against other bacteria were possible [2]. We used seropositivity for phase II IgG as an indicator for *C. burnetii* exposure, but it should not be confused with clinical illness. The timing of *C. burnetii* exposure could be better estimated if these specimens are further tested for IgM.

There is much we do not know about the epidemiology of *C. burnetii* in South Africa. This work should ideally be followed by a case–control study to improve the understanding of clinical Q fever disease in high-risk groups in South Africa; confirm/exclude risk factors associated with *C. burnetii* infection; and to study specific effective protective measures. Such research should be coupled with epidemiological investigation of *C. burnetii* in the surrounding livestock. Investigation is also needed to improve the understanding of the ecology and virulence factors of the *C. burnetii* strain(s) prevalent in the country.

## 5. Conclusions

A high level of *C. burnetii* IgG seropositivity was detected in this group of South African abattoir workers. This study suggests the workplace as a source of exposure to *C. burnetii,* and that staff spending prolonged times in contact with carcasses could be at higher risk.

## Figures and Tables

**Table 1:** Characteristics and *Coxiella burnetii* seropositivity of participants in the abattoir survey, South Africa, 2018.

Characteristic	Total Number	Proportion Seropositive (%) ^#^
Sex		
Female	104	31/100 (31.0)
Male	265	77/250 (30.8)
Education		
None	15	4/14 (28.6)
Primary school	42	11/41 (26.8)
Secondary school	294	85/278 (30.6)
Higher education	18	8/17 (47.1)
Job description		
Abattoir cleaner	15	5/15 (33.3)
Abattoir management	8	2/8 (25.0)
Abattoir workers	291	85/275 (30.9)
Other	55	16/52 (30.8)
Compulsory personal protective equipment		
Gloves	324	89/302 (29.5)
Face mask	271	80/254 (31.5)
Goggles	257	72/241 (29.9)

^#^ For those with valid test results. Individual data were missing for 13 of the 382 participants and 23 test results were equivocal.

**Table 2:** Apparent and true seroprevalence estimates for *Coxiella burnetii* in the abattoir survey, South Africa, 2018.

	Seroprevalence	95% Confidence Interval
Apparent estimate	33%	28–38%
True estimate	30%	25–35%

**Table 3:** Univariable and multivariable logistic regression analysis for factors associated with *Coxiella burnetii* seropositivity in the abattoir survey, South Africa, 2018.

Variable	Total Number	Percent Seropositive	Univariable Analysis		Multivariable Analysis	
			Unadjusted Odds Ratio (95% CI)	*p* Value	Adjusted Odds Ratio (95% CI)	*p* Value
Age			0.96 (0.93–0.98)	0.004	0.96 (0.94–0.98)	<0.001
Education				0.086 *		
Primary school	41	26.8	2.13 (0.25–18.11)			
Secondary school	278	30.6	3.68 (0.70–19.19)		Eliminated	
Higher education	17	47.1	5.25 (0.68–40.29)			
None	14	28.6	Reference			
Abattoir throughput				0.143		
High (>40 sheep/day)	332	30.7	1.62 (0.83–3.14)		Eliminated	
Low	27	25.9	Reference			
Animals sourced for personal consumption				0.011		
Yes	248	27.4	0.60 (0.41–0.87)		0.74 (0.51–1.08)	0.114
No	111	36.9	Reference		Reference	
Animals sourced direct from private farms				0.115		
Yes	316	29.7	0.62 (0.34–1.14)		Eliminated	
No	43	34.9	Reference			
Slaughter pigs				0.036		
Yes	87	24.1	0.61 (0.39–0.96)		Eliminated	
No	272	32.4	Reference			
Slaughter sheep only				0.136		
Yes	53	26.4	0.71 (0.44–1.13)		Eliminated	
No	306	31.0	Reference			
Personnel required to wear a mask				0.122		
Yes	254	31.5	1.34 (0.92–1.97)		Eliminated	
No	105	27.6	Reference			
Previous work experience				0.101 *		0.051 *
Farming	60	28.3	1.01 (0.48–2.11)		0.86 (0.43–1.68)	0.629
Abattoir/butchery	94	36.2	1.86 (0.97–3.58)		1.89 (1.13–3.17)	0.019
Other	195	28.7	Reference		Reference	
Daily duration of contact with carcass/meat products				<0.001 *		0.001 *
<half day	33	18.2	1.41 (0.24–8.09)		1.72 (0.29–10.30)	0.530
Whole day	299	33.1	3.52 (1.30–9.52)		4.65 (1.51–14.41)	0.011
<1 h/day	17	11.8	Reference		Reference	
Schedule				0.099		
Full time	332	31.0	5.06 (0.71–36.15)		Eliminated	
Part time	13	23.1	Reference			
Wearing protective clothing at work				0.142		
Regularly/always	317	31.2	1.75 (0.81–3.80)		Eliminated	
Sometimes/ never	32	25.0	Reference			
Slaughter, evisceration and/or carcass dressing				0.166		
Yes	200	32.0	1.37 (0.87–2.16)		Eliminated	
No	149	28.9	Reference			
Freezing finished products				0.035		
Yes	142	27.5	0.77 (0.61–0.98)		Eliminated	
No	207	32.9	Reference			
Transporting processed material				0.101		
Yes	93	25.8	0.67 (0.41–1.09)		Eliminated	
No	256	32.4	Reference			
Other close animal/product contact				0.071		
Yes	38	23.7	0.49 (0.23–1.07)		Eliminated	
No	311	31.5	Reference			
Treatment for any chronic illness				0.034		
Yes	89	22.5	0.68 (0.48–0.97)		0.72 (0.46–1.13)	0.139
No	259	33.6	Reference		Reference	
Livestock ownership				0.001		
Yes	54	14.8	0.34 (0.20–0.60)		0.32 (0.15–0.71)	0.008
No	295	33.6	Reference		Reference	

CI: confidence interval. The *p*-value of the whole variable (Wald test) is provided where a variable comprises more than one category (*). Univariable analysis included factors associated with seropositivity (*p* < 0.20 in likelihood ratio test). The multivariable regression model included factors associated with seropositivity (*p* < 0.10 in backward elimination), with a total of 348 observations. Model fit assessed with the goodness-of-fit test for logistic regression models fitted using survey data (*p* = 0.052).

## Data Availability

The data presented in this study have been provided in the Supplementary Materials. The data are not yet filed in a publicly accessible repository.

## Supplementary Materials

The following are available online at https://www.mdpi.com/article/10.3390/tropicalmed7020028/s1, Table S1: The serological status for *C. burnetii* and questionnaire responses for the variables analyzed in the abattoir survey, South Africa, 2018.
